# Dengue virus infection and pregnancy outcomes during the 2017 outbreak in Ouagadougou, Burkina Faso: A retrospective cohort study

**DOI:** 10.1371/journal.pone.0238431

**Published:** 2020-09-04

**Authors:** Serge Alain Tougma, W. Noélie Zoungrana/Yaméogo, Désiré Lucien Dahourou, Ida Adéline Salou/Kagoné, T. Rébeca Compaoré, Ahmed Kaboré, Thérèse Kagoné, Maxime K. Drabo, Nicolas Meda

**Affiliations:** 1 Université Joseph Ki-Zerbo, Ouagadougou, Burkina Faso; 2 Ministry of Health, Ouagadougou, Burkina Faso; 3 Tingandogo University Teaching Hospital (CHU-T), Ouagadougou, Burkina Faso; 4 Institut de Recherche en Sciences de la Santé (IRSS), Ouagadougou, Burkina Faso; 5 Centre Muraz, Bobo Dioulasso, Burkina Faso; Emory University School of Medicine, UNITED STATES

## Abstract

**Introduction:**

Dengue fever is a re-emerging pathology in Burkina Faso. It affects everyone and pregnant women are not left out. The objective of this study was to estimate the burden of dengue fever and to assess its effects on pregnancy outcomes in hospitalized pregnant women during the 2017 outbreak in Ouagadougou, Burkina Faso.

**Method:**

This was a retrospective cohort study including febrile pregnant women from five health facilities in Ouagadougou. The study was carried out from July 1st to December 31st, 2017. A logistic stepwise regression was performed to identify the pregnancy adverse outcomes risk factors.

**Results:**

Our study included 424 pregnant women at a mean age of 27.1 years old (Standard deviation: 6.23 years). Overall 28.54% (121/424) were infected with dengue virus. During follow-up, 29.01% (123/424) presented an adverse pregnancy outcome. Adjusted for gestational age and clinical symptoms, the risk of adverse pregnancy outcome was twice as high among dengue infected women as compared to uninfected women with an adjusted Odds Ratio (aOR) = 2.09 (1.08–4.05). The risk of the adverse pregnancy outcome was higher in the third trimester of pregnancy with aOR = 1.66 (1.02–2.72) in dengue fever infected women.

**Conclusion:**

Dengue fever is a risk factor for adverse pregnancy outcomes, especially in the third trimester in Burkina Faso. The implementation of effective anti-vectorial control interventions and better management of dengue fever during pregnancy are needed to improve pregnancy outcomes.

## Introduction

Globalization and the rapid urbanization of our growing cities expose people to mosquito bites which transmit the dengue virus [1, 2]. Dengue fever is a public health issue in the subtropical and tropical regions [2]. Its incidence has increased dramatically around the world in recent decades [3]. The number of dengue cases per year is estimated at 390 million, of which 96 million are symptomatic [4]. Burkina Faso has experienced in recent years a reemerging of Dengue fever [5]. It has become, like malaria, an endemic disease that adds to the burden of infectious diseases [6]. In 2017, cases of dengue fever have been identified in public and private health centers. During this outbreak, 15,096 suspected cases were reported, including 8804 probable cases, 695 confirmed cases and 30 deaths in the country according to the National Reference Laboratory for Viral Hemorrhagic Fevers (LNR-FHV). Dengue virus infection affects all socio-demographic segments of the population, and pregnant women are not left out [7].

Pregnant women are a vulnerable population for whom this infection causes more fear in relation to the outcome of pregnancy [8, 9]. Dengue fever during pregnancy could expose to more severe infection, and to mother-to-child transmission of dengue [10]. Studies on dengue fever and pregnancy have been conducted especially in Asian and Latin American regions where dengue fever has been endemic for decades. A survey of dengue fever in Guyana reported that 1.9% [95% CI: 0.9–2.9] of infected women had developed a recent infection. Among them, 92% [95% CI: 90–94] had been in contact with a flavivirus in the wild, at least once [11]. Compared with non-pregnant woman, an increase in severe dengue has been reported in pregnant women in Brazil during the second and third trimesters (OR: 3·38, 95% CI 2·10–5·42) [12]. Studies also reported fetal and neonatal complications like miscarriage, stillbirth, premature delivery, low birthweight and perinatal infection [13–16]. In a study conducted in urban areas of Ouagadougou, Burkina Faso, Collenberg et al. in 2006 found a dengue prevalence of 39.0% [17]. However, the epidemiology of dengue fever and its impact on pregnancy are not enough known, particularly in sub-Saharan Africa region, where the disease is reemerging [3]. This study aims to estimate the impact of dengue fever infection on pregnancy outcomes during the 2017 outbreak in Ouagadougou (Burkina Faso).

## Materials and methods

### Study design, sites and population

We conducted a retrospective cohort study from July 1^st^ to December 31^st^, 2017, in Ouagadougou the capital city of Burkina Faso. The rainy season occurred between May and September. Health services in Ouagadougou are provided by public health facilities including four university hospitals (third level), five district hospitals (second level), sixty primary healthcare centers; and several private healthcare centers. This study was implemented in five health facilities having a functional maternity with the capacity to hospitalize pregnant woman: Yalgado Ouedraogo University Teaching Hospital (CHU/YO), Tingandogo University Teaching Hospital (CHU-T), Bogodogo District Hospital (HDB), Saint Camille Hospital (HOSCO), Medical Centre with Surgical Services Schiphra (CMA Schiphra) and the Medical Centre with Surgical Services Paul VI (CMA Paul VI). We conducted a census of the study population in these different health facilities in the city of Ouagadougou.

All febrile pregnant women hospitalized in the study’s health facilities during the study period were eligible for this study. The inclusion criteria were: i) presenting with fever (body temperature ≥ 37.5° C) or history of self-reported fever in the last 07 days; ii) being hospitalized and followed until discharge from the hospital. Pregnant woman with unusable or non-existent medical record and those lost to follow-up during the hospitalization or discharged without medical advice were excluded.

### Collection of data

In each health facility, data were collected in the pathological pregnancy units and the emergencies of the Gynecology-Obstetrics Department. We extracted the data from the hospitalized patients and the curative consultation files. We collected sociodemographic data (the age of participant, occupation, residence, level of education, marital status), clinical data (age of pregnancy, type of pregnancy, dengue fever status), biological data and pregnancy outcomes. A patient was classified as dengue infected when she presented clinical signs of dengue fever with positive dengue Rapid Diagnosis Test SD Bioline Dengue Duo (Alere, Abbott, Sandiego, CA, USA).

### Measures

#### Study outcomes

The pregnancy outcome (positive pregnancy outcome or adverse pregnancy outcome) was the study outcome. The pregnancy outcome was defined adverse if at least one of the following events occurred: threatened abortion; spontaneous abortion, premature delivery threat, premature delivery, delivery hemorrhage, fetal death in utero, maternal death, caesarean section, obstructed labor. Otherwise, the pregnancy outcome is considered positive.

#### Data analysis

Statistical analysis was performed with the STATA software (StataCorp LP, College Station, TX, USA). The QGis 2.18.22 software was used to draw the map. Categorical data are presented as frequencies (percentage), whereas continuous variables are presented using the mean and standard deviation (SD) for normally distributed continuous data or using median and interquartile ranges (IQR) for non-normally distributed continuous data. To compare the characteristics of the study population, we used Chi-squared or Fisher’s exact tests for categorical variable and t tests for continuous variables. Factors associated with pregnancy outcome were investigated with logistic regression models. Variables with a p-value less than 0.25 in univariate analysis and missing data did not exceeding 10% were selected in a step-down procedure multivariate analysis. Variables were retained in the final model if significantly associated with pregnancy outcome (p less than 0.05).

#### Ethics

This study used data collected for clinical purposes and public health surveillance. Because of a legitimate public health purpose, the ethical approval and the informed consent was not required for this study. To assure the anonymity, we did not collect patient’s name; we used a unique identifiers.

## Results

**Fig 1:**
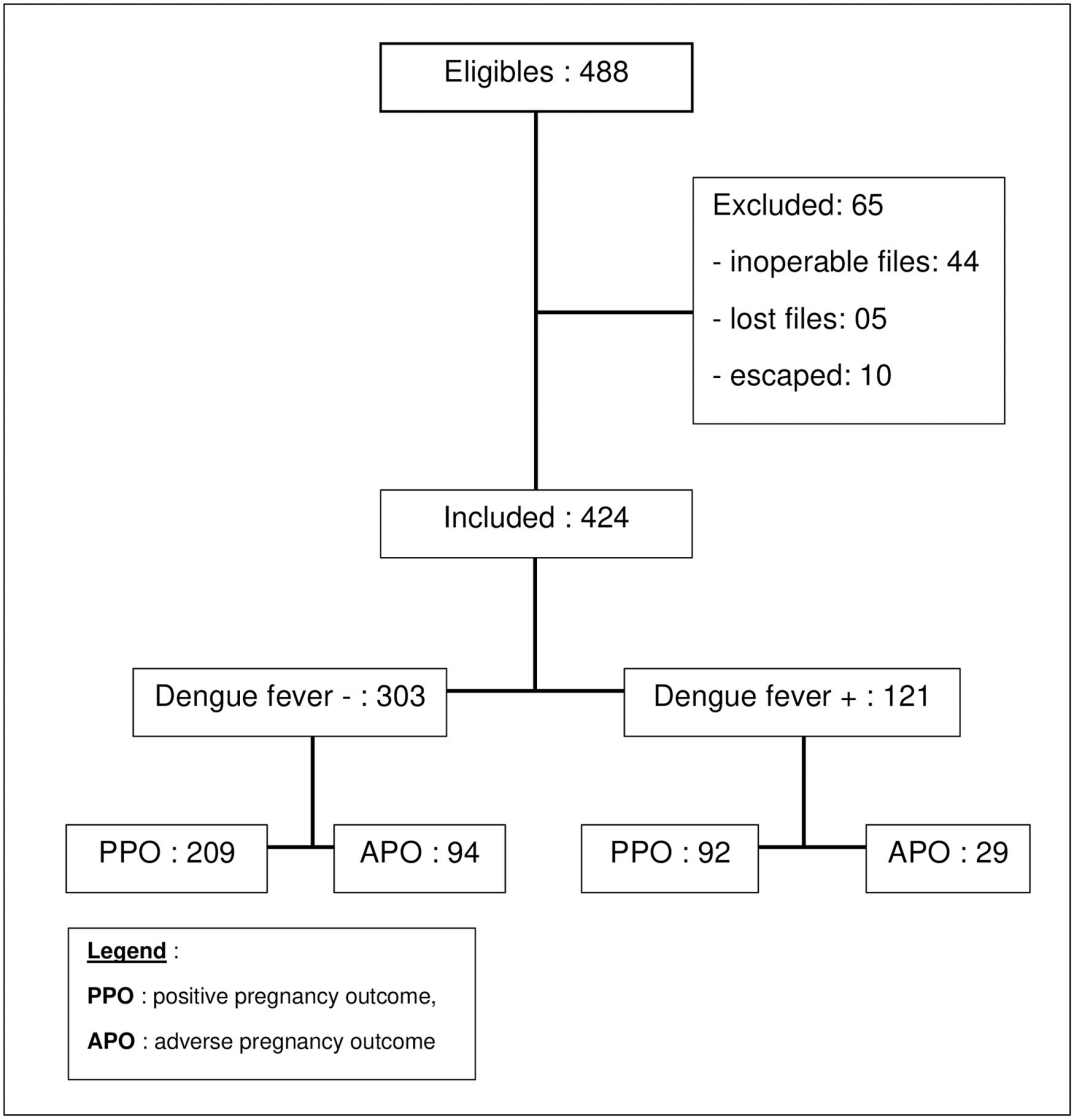
Flow chart of study participants.

From July to December 2017, 488 pregnant women with fever were hospitalized in the study health facilities. Out of them, 424 febrile pregnant women were included. (Fig 1).

Participants’ age ranged from 16 to 49 years with the mean age of 27.1 years (Standard deviation: 6.23 years). The characteristics of the participants are summarized in Table 1. The age group of 26–35 years was most represented (47.74%) in our study. The majority of the participants were housewives (45.10%), were in their third trimester of pregnancy (45.10%) and almost all carried a mono-fetal pregnancy (98.03%).

**Table 1:** Distribution of socio-demographic characteristics of participants during the 2017 epidemic in the city of Ouagadougou.

Characteristics	Frequency N (%)	Dengue fever N (%)		P value
		**No**	**Yes**	
**Age n = 421**	Mean age: 27.1 SD: 6.23			
**16–25**	178 (42.28)	115 (64.61)	63 (35.39)	
**26–35**	201 (47.74)	154 (76.62)	47 (23.38)	0.035
**36–49**	42 (9.98)	31 (73.81)	11 (26.19)	
**Age of pregnancy n = 408**				
First trimester	96 (23.53)	71 (73.96)	25 (26.04)	
Second Quarter	128 (31.37)	99 (77.34)	29 (22.66)	0.118
Third Quarter	184 (45.10)	123 (66.85)	61 (33.15)	
**Type of pregnancy n = 406**				
Single fetus	398 (98.03)	282 (70.85)	116 (29.15)	0.112
Multi-fetal	8 (1.97)	8 (100)	0 (0)	
**Profession n = 309**				
Public employee	65 (21.04)	50 (76.92)	15 (23.08)	
Private employee	53 (17.15)	36 (67.92)	17 (32.08)	0.069
Housewife	142 (45.95)	86 (60.56)	56 (39.44)	
Student / Student	49 (15.86)	37 (75.51)	12 (24.49)	
**Marital status n = 327**				
Married	252 (77.06)	166 (65.87)	86 (34.13)	0.046
Single	75 (22.94)	59 (78.67)	16 (21.33)	
**Educational level n = 137**				
Primary school	5 (3.65)	5 (100)	0 (0.00)	
Secondary school	55 (40.15)	38 (69.09)	17 (30.91)	0.072
Tertiary	60 (43.80)	51 (85.00)	9 (15.00)	
Not in school	17 (12.41)	11 (64.71)	6 (35.29)	
**Duration of hospitalization n = 414**				
[1–5]	349 (84.30)	271 (77.65)	78 (22.35)	
[6–10]	51 (12.32)	22 (43.14)	29 (56.86)	0.000
[11–15]	9 (2.17)	2 (22.22)	7 (77.78)	
[16–22]	5 (1.21)	1 (20.00)	4 (80.00)	

**Fig 2:**
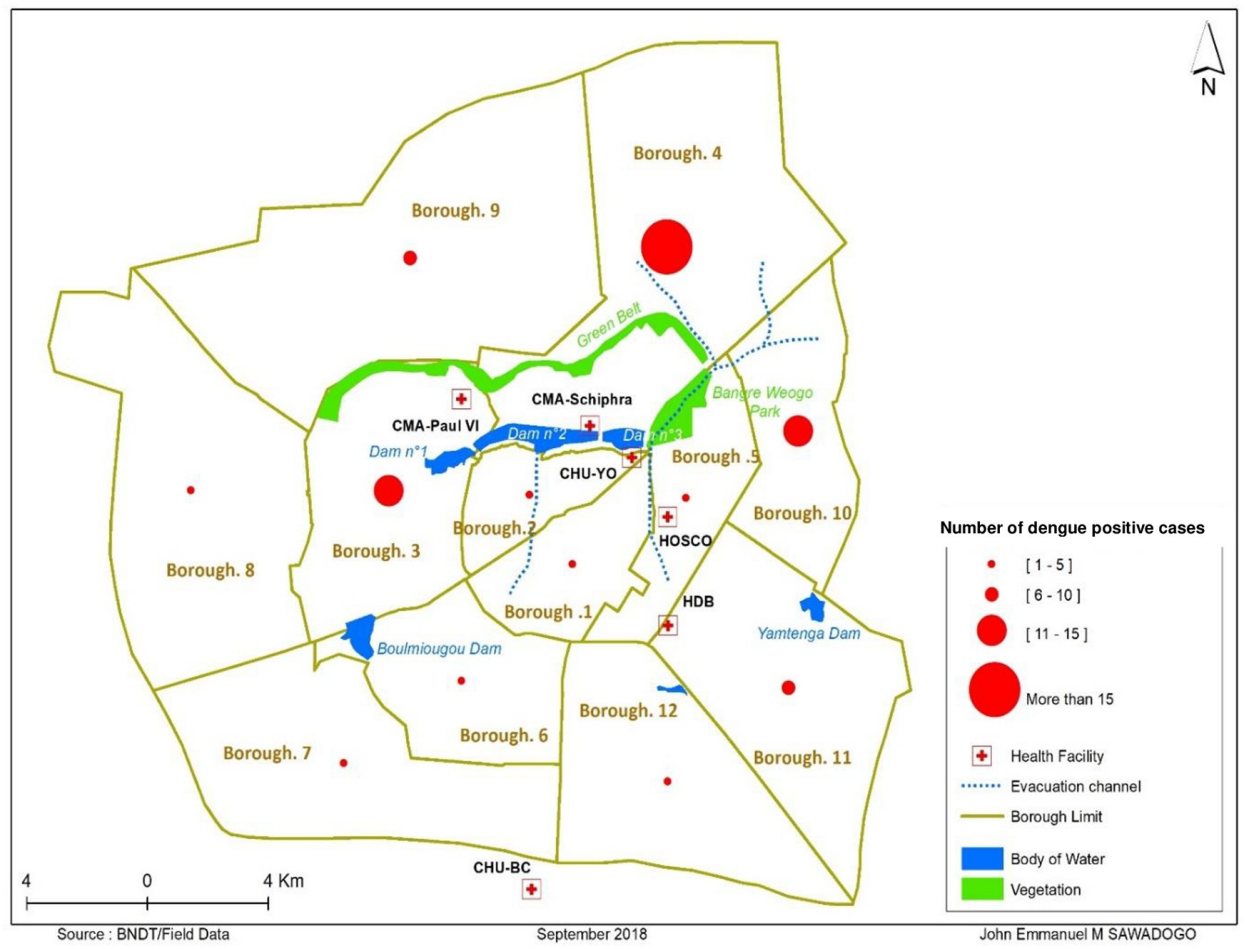
Distribution of participants by borough, vegetation, body of water and evacuation channel in the city of Ouagadougou during the 2017 epidemic.

Table 2 presents the participants’ clinical characteristics. The cumulative incidence of dengue fever infection in febrile pregnant women hospitalized in the study was 28.54% (121/424). Malaria and non-specified infectious syndrome were the most common diagnosis reported in our study with cumulative incidences of 39.86% and 30.19%, respectively. We also notify that events such as spontaneous abortion, premature delivery threat, premature delivery and maternal death were statistically associated with dengue virus infection according to Table 3. The 16 to 25 age group is the age group most affected by dengue fever in our study. The distribution of dengue fever cases in the city of Ouagadougou shows a significant concentration of infection in the districts located near dams, vegetation and gutters (Fig 2). Adverse outcome was most commonly reported in the third trimester of pregnancy in 34.78% of cases, followed by the second trimester in 25.78% of cases in our study. The association between adverse outcome and dengue fever was not statistically significant.

**Table 2:** Distribution of clinical characteristics of participants during the 2017 epidemic in the city of Ouagadougou.

Characteristics	Frequency N (%)
**Dengue fever**	
**No**	303 (71,46)
**Yes**	121 (28,54)
**Malaria**	
**No**	255 (60,14)
**Yes**	169 (39,86)
**Urinary tract infection**	
**No**	414 (97,64)
**Yes**	10 (2,36)
**Genital infection**	
**No**	421 (99,29)
**Yes**	3 (0,71)
**HIV infection**	
**No**	420 (99,06)
**Yes**	4 (0,94)
**High blood pressure**	
**No**	414 (97,64)
**Yes**	10 (2,36)
**Pre-eclampsia**	
**No**	412 (97,17)
**Yes**	12 (2,83)
**Eclampsia**	
**No**	423 (99,76)
**Yes**	1 (0,24)
**Sickle cell disease**	
**No**	418 (98,58)
**Yes**	6 (1,42)
**Diabetes**	
**No**	421 (99,29)
**Yes**	3 (0,71)
**Infectious disease syndrome**	
**No**	296 (69,81)
**Yes**	128 (30,19)

**Table 3:** Distribution of participants characteristics by dengue and non-dengue groups during the 2017 epidemic in the city of Ouagadougou.

Characterictics	Dengue Fever		p-value
	No	Yes	
**Threatened abortion n = 424**			
No	289 (71.36)	116 (28.64)	1
Yes	14 (73.68)	5 (36.32)	
**Spontaneous abortion n = 424**			
No	271 (69.49)	119 (30.51)	0.001
Yes	32 (94.12)	2 (5.88)	
**Premature delivery threat n = 424**			
No	276 (70.05)	118 (29.95)	0.02
Yes	27 (90.00)	3 (10.00)	
**Premature delivery n = 424**			
No	292 (72.82)	109 (27.18)	0.016
Yes	11 (47.83)	12 (52.17)	
**Delivery hemorrhage n = 424**			
No	302 (71.39)	121 (28.61)	1
Yes	1 (100)	0 (0.00)	
**Fetal death in utero n = 424**			
No	295 (71.60)	117 (28.40)	0.75
Yes	8 (66.67)	4 (33.33)	
**Caesarean section n = 424**			
No	288 (71.29)	116 (28.71)	0.81
Yes	15 (75.00)	5 (25.00)	
**Maternal death n = 424**			
No	302 (72.25)	116 (27.75)	0.008
Yes	1 (16.67)	5 (83.33)	

During follow-up, 29.01% (123/424) of women with fever presented an adverse pregnancy outcome. The presence of only one of these events defined an adverse pregnancy outcome: threat abortion, abortion, threat of premature delivery, premature delivery, hemorrhagic delivery, fetal death in utero, caesarean section and maternal death. Ninety four of the participants with dengue fever had an adverse pregnancy outcome.

The multivariate analysis of the risk factors associated with the occurrence of an adverse pregnancy outcome is summarized in Table 4. Adjusted for gestational age and presence of infectious disease syndrome, the risk of an adverse pregnancy outcome was significantly higher (adjusted odds ratio [aOR] = 2.09) in dengue fever infected pregnant women, women with sickle cell disease (aOR = 9.71), pre-eclampsia (aOR = 14.81) compared to uninfected pregnant women.

**Table 4:** Multivariate analysis of risk factors associated with adverse pregnancy outcomes among pregnant women during the 2017 epidemic in Ouagadougou city.

Parameter	Adverse outcome	Crude OR (CI 95%)	p-value	Adjusted OR (CI 95%)	p-value
**Dengue fever**					
No	94 (76.42)	2.19 (1.11–4.30)	0.023	2.09 (1.08–4.05)	0.028
Yes	29 (23.58)	1		1	
**Sickle cell disease**					
No	119 (96.75)	9.53 (1.41–64.10)	0.020	9.71 (1.52–62.05)	0.016
Yes	4 (3.25)	1		1	
**Pre-eclampsia**					
No	114 (92.68)	9.98 (2.22–44.69)	0.003	14.81 (3.57–61.44)	0.000
Yes	9 (7.32)	1		1	
**Infectious disease syndrome**					
No	59 (47.97)	9.19 (4.90–17.25)	0.000	8.47 (4.59–15.62)	0.000
Yes	64 (52.03)	1		1	
**Gestational age**					
First trimester	22 (18.49)	1		1	
Second quarter	33 (27.73)	1.45 (0.73–2.87)	0.276	-	
third trimester	64 (53.78)	2.07 (1.09–3.91)	0.025	1.66 (1.02–2.72)	0.041
**HBP**					
No	117 (95.12)	3.27 (0.68–15.68)	0.139		
Yes	6 (4.88)	1			
**Genital infection**					
No	421 (99.29)	3.40 (0.26–43.05)	0.343		
Yes	3 (0.71)	1			
**Age group**					
16–25	49 (39.84)	1			
26–35	55 (44.72)	0.93 (0.55–1.56)	0.801		
36–49	19 (15.45)	1.70 (0.76–3.78)	0.193		

## Discussion

The study was conducted in a context of re-emerging of dengue fever in some localities of Burkina Faso. Indeed, the country has experienced successively two major outbreaks of this pathology in 2016 and 2017 which made the country recognize as endemic.

The results may be interesting to address some concerns of clinicians, and people related to the consequences of dengue in the pregnancy. The study provided reliable data to estimate the association between dengue fever and adverse pregnancy outcome. It allowed to estimate the cumulative incidence of dengue fever in febrile pregnant women, a profile of this population and the risk of dengue fever adverse outcome on pregnancy in our context.

The cumulative incidence of dengue fever infection in febrile pregnant women hospitalized in the study was 28.54% (121/424). This cumulative incidence is lower than incidence reported by previous studies conducted in Burkina Faso [17, 18]. This difference is probably related to the inclusion of only probable cases of dengue fever in our study. However, compared with some studies conducted after 2000s which reported incidence ranged from 1.9% to 7.3% [19–21]. Our result probably overestimated this incidence. Some of these results could be due to the fact that these studies included only confirmed cases. Nevertheless, the cumulative incidence found in our study remains high because about one out of four pregnant hospitalized women presented dengue fever. Another reason is that our study used secondary data, some pregnant women with dengue fever may not have been screened or hospitalized. If nothing is done, we will witness the increase in cases of dengue and the increase in fear among this vulnerable population. In a context where the burden of malaria already weighs on their shoulders, it is imperative to increase and improve public health measures to prevent this population from being infected with the dengue virus [22]. Furthermore, the distribution of incident cases by boroughs showed high incidences in around of boroughs located near watercourses, gutters and classified forests. This situation is explained by the nature of the vector of this infection which is aquaphile and tends to develop near areas with water retention and especially during the rainy season [23]. To prevent dengue fever in this population the implementation of anti-vectorial programs in these areas is crucial.

The risk of an adverse pregnancy outcome was twice as high in febrile pregnant women infected with dengue fever compared to non-infected women. This result provides evidence that dengue fever is a risk factor for an adverse pregnancy outcome in our setting. These results confirm those of several other studies that have reported that dengue fever in pregnant women constitute a risk of pre-term birth, fetal and maternal mortality, abortion, and maternal and fetal hemorrhage. Especially if the latter occurred late during third trimester of pregnancy [11, 22, 24–28]. In terms of clinical form, severe dengue fever is implicated in these life-threatening complications due to plasma leakage, fluid accumulation, respiratory distress, profuse hemorrhages, or organ failure [2, 25]. A study conducted in 2016 based on pathophysiology demonstrated that the high risk of adverse fetal outcomes related to the production of pro-inflammatory cytokines including IL6, IL8 and TNF alpha. The latter would affect the uterus through increased production of uterine activation proteins causing premature deliveries. Thus, it would attribute fetal death in utero to the presence of the virus in the placenta causing stromal edema, an increase in syncytial nodes and chorangiosis [29]. In addition, a misdiagnosis or a late diagnosis or even a co-infection could lead to delay or poor management and thus increased the adverse outcomes [30, 19]. It is important that clinicians think about dengue fever in pregnant women with fever, to better manage dengue fever in pregnancy.

However, our study population selection criteria and the retrospective nature of this study invite us to be cautious as they might be overestimate or underestimate the burden of dengue in febrile pregnant women in Burkina Faso. Similar studies including meta-analyzes have not found a statistically significant relationship between dengue fever in pregnant women and the following adverse outcomes: preterm birth, abortion and fetal mortality [20, 31, 32]. This discrepancy could be related to the fact that our main outcome is combination of several adverse pregnancy outcomes. Another reason could be a dengue genetic susceptibility difference between African populations compared to Asian, European and Latin American populations; although this genetic susceptibility remains difficult to determine in the light of current data [28]. Nevertheless, the study highlights the need to consider dengue fever as a serious risk factors of adverse pregnancy outcomes especially in the third trimester and to implement a program focused on targeted prevention, early diagnosis, and effective management of cases and continuing education of health workers to emerging pathologies in our context [22].

## Conclusion

Dengue fever in pregnant women is a public health problem in Burkina Faso with regard to the cumulative incidence in our study where one in four hospitalized febrile pregnant women had been infected with dengue fever. It leads to twice as much risk of adverse pregnancy outcomes in infected women compared to uninfected women. It should now be considered as serious and need adequate and early management if we aim to avoid the complications related to pregnancy. Further investigations are still needed to characterize the different serotypes of the virus and the association of the infection with the adverse pregnancy outcomes. Prevention based on effective anti-vector control measures and hope for an effective future vaccine could overcome this infection in this population.

## Supporting information

S1 File (XLS)
